# “Human Babesiosis”: An Emerging Transfusion Dilemma

**DOI:** 10.1155/2012/431761

**Published:** 2012-03-28

**Authors:** Helieh S. Oz, Karin H. Westlund

**Affiliations:** ^1^Department of Physiology, University of Kentucky Medical Center, Lexington, KY 40536, USA; ^2^Department of Internal Medicine, University of Kentucky Medical Center, Lexington, KY 40536, USA

## Abstract

Babesiosis, a common disease of animals, can infect humans via vector “tick bite”, particularly in endemic areas. The recent reports of fatal cases in Hepatitis C and postliver transplant patients resulting from transfusion of contaminated blood should alert the medical profession regarding this emerging dilemma in endemic as well as nonendemic areas and the need for accurate blood screening for transfusion. Here, we illustrate different stages of the parasite lifecycle, progression of babesiosis in animal model, some aspects of pathologic outcomes, ongoing therapeutic modalities, and a feasible Acridine Orange fluorescent methodology for the diagnostic evaluation of blood samples.

## 1. Introduction

**Figure 1:**
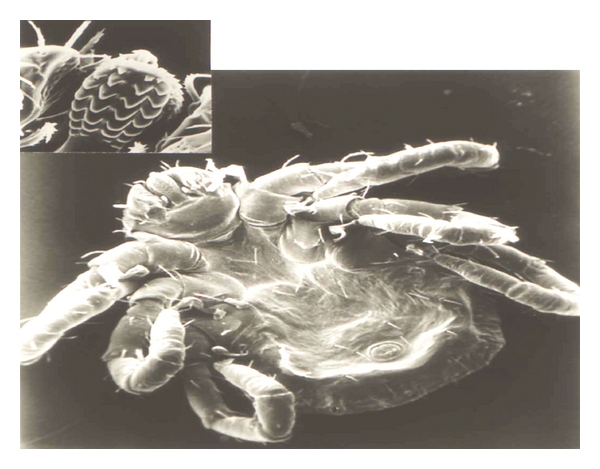
Scanning electron micrograph of an *Ixodes* tick larva with enlarged mouth part (mandibles and hypostome) demonstrating blade-shaped denticules. Tick inserts denticles into the flesh of the victim, opens them like an umbrella during blood meal, and infects the host with *babesia* (sporozoites).

Human babesiosis, is transmitted by the bite of species “*Ixodes*” tick (Figure 1) or by the blood transfusion from an infected individual in North America [1, 2]. Isolated reports have been documented of possible transplacental or congenital transmission of babesiosis to neonates [3]. In USA, clinical cases of babesiosis have been reported primarily in endemic areas, in the Northeast (New England and New York) to Midwest (Wisconsin and Minnesota), and California [4–7]. Recent cases from nonendemic area provide an alert to the medical professions including hepatology and gastroenterology community regarding emergence of the potential for transfusing contaminated blood and blood products with this infectious agent [7–9]. This includes fatal incident of babesiosis in two patients with the history of Hepatitis C, blood transfusion, and/or liver transplantation. Patient a, 43-year-old female from a nonendemic area (Delaware) was admitted after 3 days of fever, cough, and fatigue. The patient had been treated with pegylated interferon for 40 weeks and was blood-transfusion-dependent for the most of her life. She was identified with babesiosis allegedly resulting from an infection with *Babesia microti* (*B. microti*) after receiving a contaminated blood transfusion. She was treated with clindamycin/quinine, but patient expired 3 days after admission [7, 10]. The second case was a 73-year-old female from Indiana with the history of liver transplant, splenectomy, and blood transfusion. She was admitted for fever, dark urine, jaundice, and identified with 6% parasitemia. She was treated with a clindamycin/quinine combination and additional blood transfusion with no improvement, and increased parasitemia (50%). The patient expired 2 days later [8]. Additionally, 57-year-old female patient with a 26-years history of Crohn's disease and from endemic area (Cape Cod, MA) developed a severe fever (102°F) and syncope. She was on antitumor necrotic factor (anti-TNF*α*: Infliximab, Remicade) maintenance therapy (5 mg/kg every 8 weeks) for a period of 10 months and a history of tick bites. This patient was diagnosed with 15% parasitemia and was treated successfully with atovaquone/azithromycin combination that responded favorably and recovered [6].

## 2. *Babesia microti (B. microti)*

**Figure 2:**
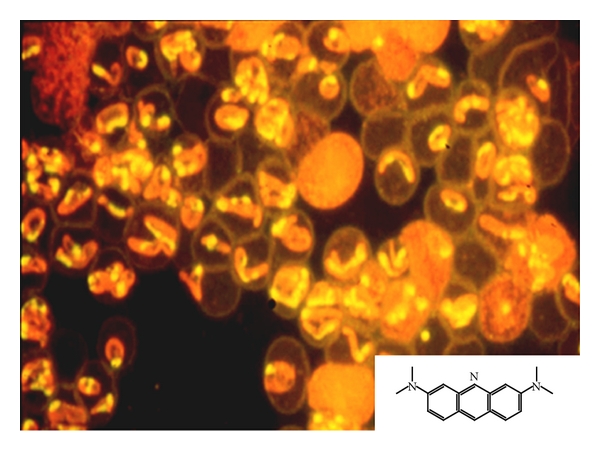
Blood smear, from hamster infected with *B. microti,* stained with acridine orange (molecular formula) and examined with fluorescent microscopy. Babesial organisms appear as single-ring forms (Trophozoites) or multinuclei (Merozoites) within the red blood cells (RBCs). Nuclei (DNA) stain yellow and the rest of the protein orange red inside the RBCs. RBCs appear as hollow ghost cells.

**Figure 3:**
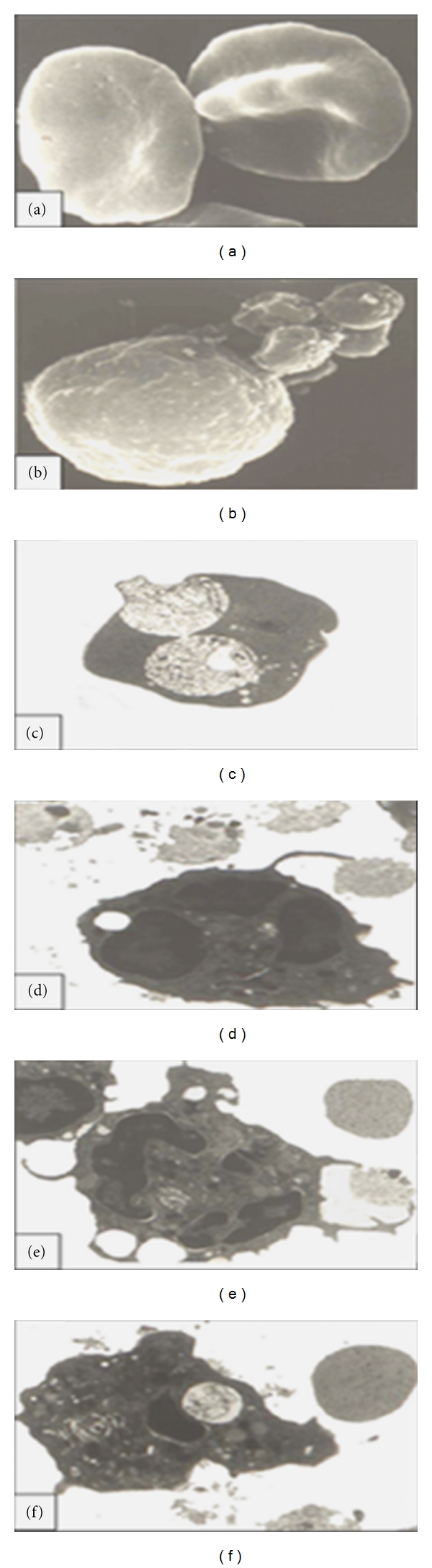
(a, b) are scanning electron micrographs and (c–f) transmission electron micrographs from the cross-sections. (a) infected RBCs from hamster harboring babesial organisms. (b) immune cell (neutrophil) attached to babesial organisms via extended pseudopods. (c) Cross section of the infected RBC from hamster harboring 2 distinct trophozoites. (d–f) cross-section of neutrophil extending pseudopods toward extracellular *babesia* during attachment (d), engulfment (e), and ingestion (f) of the organism (hamster blood).

**Figure 4:**
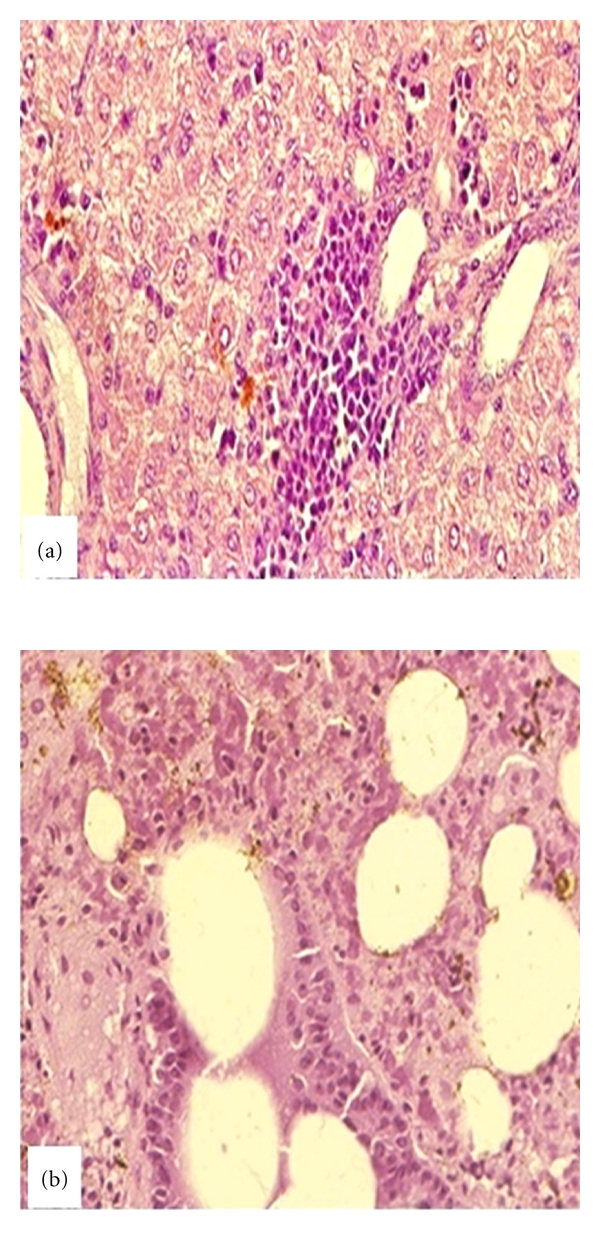
Histopathology sections from infected hamsters stained with hematoxylin and eosin. (a) Hepatitis: Hepatic section demonstrates degeneration of hepatocytes with diffuse periportal infiltration of inflammatory cells and bile stasis (brown spots) in parenchyma. (b) Pneumonitis: pulmonary section with severe edematous, diffuse infiltration of inflammatory cells, and hemosiderin deposits (brown spots) into parenchyma.

*Babesia,* is an intraerythrocytic protozoan parasite (Figures 2 and 3, blood from experimentally infected hamster with *B. microti*) which can cause a spectrum of flu-like symptoms, including benign headache and fever, in immunocompetent patients. However, in neonates, the elderly, splenectomised, immunocompromised, and AIDS patients [6–14] as well as animals [15–18], the disease can manifest with hepatitis (Figure 4(a), experimentally infected hamster), hydrothorax, pneumonia (Figure 4(b), infected hamster), myocarditis, splenomegaly, glomerulonephritis, hematuria, and hemolytic anemia resulting from fulminating babesiosis including fatal outcomes if not properly diagnosed and treated [6–8, 10, 15]. Babesiosis is named after Victor Babes, a Romanian microbiologist, who discovered the protozoan parasite in the cattle with febrile hemoglobinuria (1888).

## 3. Transmission

*Babesia* has a life cycle similar to malarial organisms with sexual stage in the vector *Ixodes* ticks (Figure 1) which transmit the organism. The sexual stage occurs in the tick salivary glands as extracellular sporozoites and sporoblasts. During a blood meal, the infested tick inserts its denticles into the flesh of the victim, opens them like an umbrella, and infects the host with sporozoites form. Rodents serve as reservoir host, and humans become accidental host when exposed to the infested tick in an endemic area. After an infected tick bite, babesial organisms enter the blood stream and invade the red blood cells (RBCs) and replicate asexual form as merozoites (Figures 2, 3(a), and 3(c), blood from infected hamster) and damage the organs. Parasites and infected RBCs harboring babesial organisms are picked up by the body immune cells, including neutrophils (Figures 3(b), 3(d), 3(e), and 3(f), blood from infected hamster), and macrophages and transferred to the splenic tissue which eventually become overwhelmed. Man can transmit the parasite by the blood transfusion, fetal congenital transmission, or infected organ transplantation [1–4, 15–18].

## 4. Animal Models

Hamster is a common animal model to study the disease and to explore drug therapy. Mice usually develop a low-grade parasitemia and not favored as a model. Hamsters are inoculated via *i.p* with an infected blood-harboring babesial organisms. The disease is usually subacute in hamsters, and peak parasitemia occurs in 2-3 weeks although infections may prove to be fatal in about l-2 months after inoculation [16]. Another hamster model was established by *B. microti* passaged into immunocompromised animals and then transitioned into immunocompetent (normal) golden hamsters [15]. Hamsters were immunosuppressed with one injection of Depo-Medrol followed by dexamethasone in daily drinking water. Then the hamsters were inoculated *i.p* with the infected blood. This model was found remarkably similar to the immunocompromised and severe cases of humans with babesiosis. Similar to acute cases in human, parasitemia raised rapidly to 70–90% in about 10 days following the inoculation. The tropism of the organism, histology, pathogenesis, and response to drugs in this fulminating hamster model was almost identical and mimicked those parameters in humans [15, 17].

## 5. Therapies

The clindamycin/quinine combination therapy has been described in the clearance of parasitemia and resolution of this disease, although, failures of this treatment approach have been reported [1, 2, 7–11, 13, 19]. In addition, blood exchange in severe cases has been shown with a variable success [7, 8, 10]. Previous studies described the efficacy of azithromycin/quinine against *B. microti* in a hamster model as a possible alternative for the clindamycin/quinine combination [16].

 Furthermore, atovaquone was described to be more effective than the combination of clindamycin/quinine in prevention as well as therapeutic babesiosis in experimental studies [17, 18]. In contrast, proguanil (an antimalarial agent) alone had no effect against the disease in the model [17, 18]. Currently, atovaquone and azithromycin combination have been reported effective in babesiosis patients from endemic area [6, 12] as well as those acquired infection via blood transfusion [1, 7, 20] including neonates [21].

Atovaquone is a 1,4-hydroxynaphthoquinone compound, an analog of ubiquinone, with potent antimalarial and antipneumocystic activity that has been effective in animals and humans for preventive and therapeutic management of *Pneumocystis carinii* pneumonitis [22–25], Toxoplasmosis [26], but not cryptosporidiosis [25]. Atovaquone has been reported, as generally, safe, well tolerated with minor side effects, and an effective alternative therapy compared to the clindamycin/quinine combination against babesiosis in cases [3, 6, 12]. More effective and better-tolerated therapeutic modalities are still needed to prevent possible development of drug resistance in the future.

## 6. Diagnosis

Patients are usually found to be asplenic, anemic with fever, and jaundice. Laboratory tests include low hemoglobin, high total Bilirubin, aspartate aminotransferase (200–7000 IU/L), creatinine levels, and hemoglobinuria [6–8, 10].

Currently Babesiosis is diagnosed using immunofluorescent assays, enzyme immunoassay EIA, ELISA, RT-PCR, and microarrays. *Babesial *immunoglobulin (IgG and IgM titers by immunofluorescent antibody (IFA) is somehow specific, and nested polymerase chain reaction (PCR) and real time to be more sensitive.

Then each case is confirmed using direct blood staining with Giemsa or Wright staining. For Giemsa staining, thin blood smears are fixed in methanol and stained for 20 min in 0.5% Giemsa stock solution in 4% PBS. The acridine orange technique (Figure 4) was found to be rapid and to provide a feasible tool and better visualization of *B. microti* in blood smears than other direct staining methods. For Acridine orange staining, blood smears are fixed in methanol and stained in 0.1% of fluorescent dye diluted in Krebs Ringer-phosphate solution pH 7.4 and then examined 10 min later by fluorescent microscopy [15]. While Giemsa-stained slides are more stable and can be stored without degradation or bleaching over long periods, the fluorescent dyes do quench; however, slides stained with fluorescent dyes can be restained at a later time with minimal loss of discriminatory power [15]. Acridine orange is a cell-permeable dye, originally extracted from coal tar and creosote oil. It is a nucleic acid selective fluorescent cationic dye that interacts and binds to DNA of the parasite with a bright yellow-green light excitation. Acridine orange also sequesters protein compartments in the cell and becomes protonated to emit orange light under fluorescent light activation. Acridine orange technique was found to be a simple and effective diagnostic tool to confirm babesial infection compared to other traditionally used direct microscopic examination techniques, for example, Giemsa and Wright's stain [15, 18].

Additionally, differential diagnosis of babesiosis should be performed with lyme disease, malaria, and other blood-borne diseases using serology, PCR, and direct microscopic evaluations.

In conclusion, the awareness of the community for a possible babesial infection, implementation of additional diagnostic techniques, improved preventive measures, vector control to interfere with the parasite life cycle, blood and blood product sanitation, broader screening of the blood donors and/or blood for transfusion, and effective drug development should be combined to prevent increases in babesial infection in endemic as well as nonendemic areas.

## References

[1] Gubernot DM, Lucey CT, Lee KC, Conley GB, Holness LG, Wise RP (2009). Babesia infection through blood transfusions: reports received by the US Food and Drug Administration, 1997–2007. *Clinical Infectious Diseases*.

[2] Krause PJ (2003). Babesiosis diagnosis and treatment. *Vector Borne and Zoonotic Diseases*.

[3] Sethi S, Alcid D, Kesarwala H, Tolan RW (2009). Probable congenital babesiosis in infant, New Jersey, USA. *Emerging Infectious Diseases*.

[4] Spaete J, Patrozou E, Rich JD, Sweeney JD (2009). Red cell exchange transfusion for babesiosis in Rhode Island. *Journal of Clinical Apheresis*.

[5] Froberg MK, Dannen D, Bernier N, Shieh WJ, Guarner J, Zaki S (2008). Case report: spontaneous splenic rupture during acute parasitemia of *Babesia microti*. *Annals of Clinical and Laboratory Science*.

[6] Cullen G, Sands BE, Yajnik V (2010). Babesiosis in a patient on infliximab for Crohn’s disease. *Inflammatory Bowel Diseases*.

[7] Van N, Civen R (2009). Babesiosis acquired through blood transfusion, California, USA. *Emerging Infectious Diseases*.

[8] Berman KH, Blue DE, Smith DS, Kwo PY, Liangpunsakul S (2009). Fatal case of babesiosis in postliver transplant patient. *Transplantation*.

[9] Wudhikarn K, Perry EH, Kemperman M, Jensen KA, Kline SE (2011). Transfusion-transmitted babesiosis in an immunocompromised patient: a case report and review. *American Journal of Medicine*.

[10] Zhao Y, Love KR, Hall SW, Beardell FV (2009). A fatal case of transfusion-transmitted babesiosis in the State of Delaware. *Transfusion*.

[11] Center of Disease Control (1983). Clindamycin and quinine treatment for *Babesia microti* infections. *Morbidity and Mortality Weekly Report*.

[12] Vannier E, Krause PJ (2009). Update on babesiosis. *Interdisciplinary Perspectives on Infectious Diseases*.

[13] Golightly LM, Hirschhorn LR, Weller PF (1989). Fever and headache in a splenectomized woman. *Reviews of Infectious Diseases*.

[14] Zaidi S, Singer C (2002). Gastrointestinal and hepatic manifestations of tickborne diseases in the United States. *Clinical Infectious Diseases*.

[15] Oz HS, Hughes WT (1996). Acute fulminating babesiosis in hamsters infected with *Babesia microti*. *International Journal for Parasitology*.

[16] Weiss LM, Wittner M, Wasserman S, Oz HS, Retsema J, Tanowitz HB (1993). Efficacy of azithromycin for treating *Babesia microti* infection in the hamster model. *Journal of Infectious Diseases*.

[17] Hughes WT, Oz HS (1995). Successful prevention and treatment of babesiosis with atovaquone. *Journal of Infectious Diseases*.

[18] Hughes W, Oz HS, Keusch G (1997). *Successful Prevention & Treatment of Babesiosis with Atovaquone. The Best of Infectious Diseases*.

[19] Persing DH, Herwaldt BL, Glaser C (1995). Infection with a *Babesia* organism in Northern California. *The New England Journal of Medicine*.

[20] Semel ME, Tavakkolizadeh A, Gates JD (2009). Babesiosis in the immediate postoperative period after splenectomy for trauma. *Surgical Infections*.

[21] Raju M, Salazar JC, Leopold H, Krause PJ (2007). Atovaquone and azithromycin treatment for babesiosis in an infant. *Pediatric Infectious Disease Journal*.

[22] Hughes W, Leoung G, Kramer F (1993). Comparison of atovaquone (566C80) with trimethoprim-sulfamethoxazole to treat *Pneumocystis carinii pneumonia* in patients with AIDS. *The New England Journal of Medicine*.

[23] Oz HS, Hughes WT, Rehg JE (1997). Efficacy of lasalocid against murine *Pneumocystis carinii pneumonitis*. *Antimicrobial Agents and Chemotherapy*.

[24] Hughes WT, Killmar JT, Oz HS (1994). Relative potency of 10 drugs with anti-*Pneumocystis carinii* activity in an animal model. *Journal of Infectious Diseases*.

[25] Oz HS, Hughes WT, Rehg JE (1999). Rat model for dual opportunistic pathogen prophylaxis: cryptosporidium parvum and *Pneumocystis carinii*. *Laboratory Animal Science*.

[26] Petersen E (2007). Toxoplasmosis. *Seminars in Fetal and Neonatal Medicine*.

